# Exploring issues in the conduct of website searching and other online sources for systematic reviews: how can we be systematic?

**DOI:** 10.1186/s13643-016-0371-9

**Published:** 2016-11-15

**Authors:** Claire Stansfield, Kelly Dickson, Mukdarut Bangpan

**Affiliations:** Evidence for Policy and Practice Information and Coordinating (EPPI)-Centre, Social Science Research Unit, UCL Institute of Education, University College London, 18 Woburn Square, London, WC1H 0NR UK

**Keywords:** Systematic reviews, Information retrieval, Website searching, Online searching, Information management

## Abstract

Websites and online resources outside academic bibliographic databases can be significant sources for identifying literature, though there are challenges in searching and managing the results. These are pertinent to systematic reviews that are underpinned by principles of transparency, accountability and reproducibility. We consider how the conduct of searching these resources can be compatible with the principles of a systematic search. We present an approach to address some of the challenges. This is particularly relevant when websites are relied upon to identify important literature for a review. We recommend considering the process as three stages and having a considered rationale and sufficient recordkeeping at each stage that balances transparency with practicality of purpose. Advances in technology and recommendations for website providers are briefly discussed.

## Background

Many systematic reviews use topic-specific bibliographic databases to identify literature in a ‘systematic’ way. The functionality of these databases facilitates highly structured Boolean searching, automated recording of search history and bulk exporting of results. These functions support transparency, accountability and reproducibility of the search process, in line with accepted principles of literature searches for systematic reviews [1, 2]. However, literature is often sought outside of bibliographic databases, regardless of subject discipline or methodological focus of the review. Approaches might involve searching websites, search engines or online repositories and typically require searching and browsing (reading and navigating) techniques that differ from approaches to searching bibliographic databases. In comparison with bibliographic databases, there are greater challenges in deciding which websites and online resources to use, running complex searches, exporting search results and documenting the process. Problems encountered when searching websites with limited search functionality include large search outputs, empirical research hidden on websites within a wealth of other material and lack of abstracts [3]. Where websites are relied upon to identify important literature for a review, it raises the issue of how the search is transparent, accountable and reproducible.

Our focus is on websites and online resources outside academic bibliographic databases. We use the term ‘websites’ in a broad sense to refer to online resources that lack the functionality to carry out complex Boolean searches, or export results, or do not readily provide a search history. Such resources vary widely in terms of appearance, functionality and content. They include websites of organisations, institutional repositories, research registers, online library catalogues and internet search engines. The value and rationale for utilising these ﻿resources varies between reviews and within review teams. Other complementary searching approaches include asking key contacts and authors, hand-searching journals, cited-reference searching and checking references.

We previously observed that relevant literature for low- and middle-income countries, such as working and policy papers, is often not included in databases, and is located from organisational websites, contacting authors or internet search engines [3]. For some systematic reviews undertaken at the Evidence for Policy and Practice Information and Co-ordinating Centre (EPPI-Centre), over a quarter of relevant citations were found from websites and internet search engines [4–13]. This finding is based on data from eight systematic reviews, of which four concern interventions in international development [4, 7, 12, 13], and four concern people’s views to inform to UK public health policy initiatives [5, 6, 8, 9].

While there is established guidance on conducting systematic searches of bibliographic databases, it is less clear how to approach searching websites for systematic reviews. We briefly describe our approach in Brunton et al. [14] and expand on this approach here. There is some specific guidance on web-searching for systematic reviews published by the Centre for Environmental Evidence [15], with emphasis on using search engines. Other related work is framed around searching for ‘grey literature’, where the aim is to seek out relevant literature that is not published in academic journals. Haddaway and Bayliss [16] consider grey literature in two forms: unpublished academic research and research that is generated by practitioners. They present different scenarios for undertaking searching for grey literature and suggest resources for each scenario. There are case studies demonstrating approaches in undertaking grey literature searching within public health-related topics published by Godin et al. [17], Mahood et al. [18] and McGrath et al. [19]. Eysenbach et al. [20] provide an approach to internet searching for unpublished clinical trials. There are published studies on searching specific resources systematically, for example, Google Scholar [21–24] and trials registers [25]. Outside of the systematic review literature, Blakeman [26] outlines challenges and approaches for searching Google effectively and discusses other online resources and tools for retrieving research.

The aim of this discussion is to: consider the challenges of searching websites and online resources outside academic bibliographic databases; to present an approach for conducting website searching for a systematic review; and consider how identifying literature from websites can be systematic in terms of being transparent, accountable, and reproducible. All the authors of this discussion have undertaken searching for systematic reviews across the fields of health systems and social care, public health, education, social policy and international development. Our approach draws on our experience from conducting systematic reviews, and supporting other review teams to undertake systematic reviews over many years and is informed by discussions within our research centre. We suggest website searching should not be considered only in relation to ‘grey literature searching’ because it can be used as a strategy to identify journal articles not identified from traditional bibliographic database searches. It might also be used to discover journal citations missed by a database search ﻿strategy, to compensate for poor access to subscription databases and for journals that are not indexed within any of the databases searched. We propose a systematic approach to the design and conduct of website searching and a method of recordkeeping. It is not our intention to describe methods for using specific resources. Neither is it to encompass reporting the search in a written published report, which is an area for separate consideration, for example, Briscoe [27] explored the reporting of how websites and search engines were searched in health technology assessments. We reflect on our approach in light of other published works, the potential implications of new technologies and make recommendations for website providers. We hope to promote further discussion of methods in literature searching for systematic reviews and other types of evidence syntheses.

### How can we be systematic?

Key challenges we encounter when searching websites for systematic reviews are (1) identifying and deciding which resources to search, (2) how to search or navigate them appropriately, (3) assessing the results, (4) deciding which literature to collect from each resource, (5) retrieving relevant literature in a usable format and (6) deciding what information to record for transparency. To help address these challenges, we propose engaging with searching websites for systematic reviews as three stages: (1) planning the search, (2) executing the search and (3) screening records for relevance and managing the results. These stages are distinct aspects that could be used to approach any type of search (e.g. contacting authors, bibliographic database searching, citation searching, website searching). Table 1 outlines the objective for each stage and challenges for undertaking this step when searching websites. We discuss each of these stages in turn, starting with a discussion on the objective of each stage and ways to address the challenges. Overall, many challenges can be addressed by considering the rationale of the approach and having sufficient recordkeeping at each stage to provide some transparency and increased rigour of approach, without the process becoming unnecessarily onerous for its purpose. We consider principles of transparency, accountability and reproducibility within each stage.

**Table 1:** Three stages within systematic searches and challenges for website searches

Stage	Objective	Challenges in undertaking this step for websites and online resources
Planning the search	• To have a rationale for searching methods, based on the purpose of the search • Planning where to search, who is undertaking the search and the timeframe of the review	• Discovery of suitable websites to use • Deciding how representative the range of websites need to be in relation to the scope of the review and time available to search
Executing the search	• To utilise each resource in a consistent way, and in a way that is appropriate for each individual resource	• Planning how to search when each source is structured differently and may differ in terms of focus and content • Using individual approaches for each website • Searching resources where the functionality for searches consisting of multiple words and Boolean searching is often limited
Screening and information management	• To assess literature for relevance (screening or sifting) • To quantify how many items of literature is processed • To report on methods	• Which literature to collect and how much screening to carry out • Limited functionality to transfer results to citation management tools • The level of detail needed for recordkeeping for preliminary screening at source

#### Planning the search

Planning the search involves having a rationale to justify and inform decisions on where to search. It also considers who is undertaking the search and the timeframe and resources available for the review. The role and purpose of website searching compared with other methods of identifying literature informs these decisions. One challenge is knowing about the most appropriate websites to search. Unlike selecting bibliographic databases, which often cover broad topic areas and specific disciplines, identifying appropriate websites is more dependent on the precise nature of the research question and knowledge and accessibility of the websites available. There is a vast range of options that vary in scope, functionality for searching and browsing and volume of content. The choice of websites should reflect those most suitable to the review, and includes deciding how representative of the topic of investigation it needs to be. There is potential for introducing unintentional bias; for example, a review covering low- and middle-income countries worldwide involves searching a combination of websites that span relevant geographical areas, and is not limited to one geographical region. Another bias could be introduced by focussing only on sources relating to a particular stakeholder group, age group, setting, or study design without appropriate reasons. It can take considerable time to search individual websites, particularly those of individual organisations, or those that contain long publication lists. A risk is that the process will not yield any unique or relevant records compared with other searching techniques, and time is spent looking at references discovered elsewhere.

To address these issues when planning the search, some understanding of the resources within a topic area is needed and can be gained in a variety of ways, by consulting methods guidance for undertaking systematic reviews; library resource lists; grey literature resource lists; reports of systematic reviews; topic advisers and internet search engines or already known websites of interest. Godin et al. [15] describe an approach where they used a series of Google searches to identify 77 relevant organisations and websites. They also used established customised Google search engines, which restrict searching to specific websites; however, some of these only display a small number of the overall search results. Some of the resources chosen will depend on the reason for website searching. Table 2 gives some examples of choosing websites for different reviews. Carefully thinking about different types of websites can help mitigate unintentional biases and limitations can be acknowledged within a search plan. Planning could involve categorising websites in terms of different characteristics such as population focus, geographical coverage, types of literature and study designs covered. Such categorising aids thinking and aids identifying gaps and limitations. Although decisions on where to search may be made at the outset of a review, these could change during the reviewing process if new resources are identified or if it emerges that some resources are not useful or are unwieldy to use.

**Table 2:** Examples of choosing websites for different reviews

Systematic review	Key purpose of website search	Types of websites, online resources and depositories
Access to economic assets for women in low- and lower-middle-income countries [7]	Discover relevant research missed or not indexed in international or regional databases	Over 35 sites consisting of government and research-active non-governmental organisations, academic research centres and funders, relating to economics, microfinance, international development, or regional development banks
Adult cooking skills programmes [31]	Discover unpublished evaluations of cooking skills programmes in the UK	Generic search engine, library catalogues, and 25 websites of UK public health and community organisations, research centres and government departments
Depression, anxiety, pain and quality of life in people living with chronic hepatitis C [32]	Discover research identified by advocacy organisations and health research potentially missed by database searches	Websites of hepatitis C advocacy groups in mainly in the UK and some resources to containing healthcare research in general
Realist synthesis of school accountability in low- and middle-income countries [33]	Undertake purposive searching for advisory group engagement and scoping exercise stages prior to bibliographic database searching	Specialist databases, search engine, 20 websites of international development agencies and organisations
Exercise interventions and patient beliefs for people with chronic hip and knee pain [34]	Discover literature on people’s experiences, largely unpublished in journals	Range of website resources covering: arthritis groups in UK, Australasia, and North America, ageing care registries, patient experience resource, grey literature resources, generic search engine, social science research

It is important to consider which review team member will undertake the searches and ensure they have sufficient understanding of the type of information that is being sought from the literature search, as well as skills in locating and managing literature found from the websites. If a review team has a policy of screening publications for eligibility by two people, they need to decide whether to extend this for website searching, and their rationale for doing so; for example, if the aim is for consistency or to help ensure relevant items are not missed. Given the potential variation by individuals in searching websites, it seems easier to operationalise the latter, without striving to match the exact process used by each person.

Another aspect to consider is the time-point of website searching alongside the rest of the systematic review. For example, if the website search is undertaken by a reviewer who has already screened literature against the eligibility criteria of the review, they would have a clear idea of the literature sought from websites. Hammerstrom et al. [1] suggest that completing web searches towards the end of the search phase of a review ensures picking up the most current information. On the other hand, searches undertaken at an early stage of the review may inform any bibliographic database searching or other searching techniques that might be planned later. Overall, we think that the searching could take place at any point, depending on what is needed for an individual review.

#### Executing the search

The objective of searching should be to utilise each resource in a consistent way and in a way that is appropriate for each individual resource. This poses particular challenges for websites because each resource is structured differently and may differ in terms of focus, content and functionality for searching and browsing. It is difficult to judge how to search with confidence that items of interest have not been missed. It is impossible, and potentially unhelpful, to treat each resource in the same way. Each website requires different techniques, for example, browsing relevant web pages, searching using a generic search function, navigating headings within webpages or scanning lists of references. It is likely that more than one approach is needed for each website, and time is needed to develop knowledge and skills to utilise individual resources.

From our experience, the process of recording how a website is searched helps in considering the search approach. The act of recording which navigation headings are browsed and which search terms are used helps the searcher to reflect on their choice and rationale of approach and may prompt useful iteration of searches. It aids structuring a search for each website, encourages a greater level of care to be taken when searching and enables comparison across different websites. Recordkeeping may help in using a consistent approach for similar resources, while at the same time giving flexibility to search each resource differently, as needed. As well as facilitating searching, such recordkeeping provides a degree of transparency and aids accountability and reproducibility for internal documentation. It also enables knowledge and skills gained from using particular websites to inform future searches, for example, if revisiting the same websites at a later date to search using different terms or to update the original search.

We recommend considered recordkeeping with brief descriptions of the techniques used for searching. Table 3 gives an example of recordkeeping in an Excel worksheet. The focus is on recording key elements quickly and efficiently in a way that is understood by the review team. Individuals may have their preferred notations for brevity. The example illustrates a range of approaches taken to identify research on a website of the Alzheimer's Society, a UK research and support charity for dementia. This includes a brief description of the date searched (and last searched, if different), the pathways followed, any search terms used and database fields searched. The notes field provides space for recording additional information. The second resource searched is Rehabdata, an online database on disability and rehabilitation. As well as recording the pathway searched, it is noted that predefined keywords from that resource were used. The uniform resource locators (URLs) for the main websites are recorded in a separate worksheet, but the specific pages can also be recorded within the section on the pathway followed. The worksheet also provides space to specify how many literature citations were browsed and saved for further examination, which is discussed under the third stage of screening and information management.

**Table 3:** Example of recordkeeping for executing the search

Name of resource	Searcher	Date searched	Date of last access (if different)	Pathway followed, e.g. browsed headings/searched site/database within website (use separate lines for the different types of searches)	Used predefined keywords ‘Y’	Notes
Alzheimer’s Society Research https://www.alzheimers.org.uk/research	CS	3/3/15		Browsed ongoing and completed research on the topic of ‘Towards better care for people with dementia’		
		3/3/15		browsed ‘living with dementia’ research articles		
		3/3/15		Searched dementia catalogue—subject: hospital admissions, hospital discharges	Y	
		3/3/15		Searched dementia catalogue-free text: transition, transitions or transfer		
Rehabdata	CS	3/3/15		Searched descriptor field: Hospitals AND (Psychiatry OR Psychiatric Rehabilitation) AND 1999–2015	Y	

We consider the process of searching and browsing as iterative, as the content within each website might prompt using different search terms, or browsing other parts of a website. Some searches may incorporate a full-text search of a document, which could require adapting search terms to increase the relevance of results. It is important to be aware of user bias in terminology and to use the headings or index terms set by the website provider or consider browsing records to complement using search terms. Establishing a pool of terms to draw on or reflecting on searches for similar websites can help.

Actual searching and browsing methods are likely to differ from review to review. For example, some review teams may adopt a uniform method to apply to specific groups of resources. On the other hand, it may be more appropriate to adopt varying approaches that are individual to a website, but consistent with the overall premise of finding relevant research that meet the criteria for a review. Depending on the content of the resource and how it is structured, it may be appropriate to browse references, rather than run searches on keywords, or use a combination of approaches. Searching some registers or websites focussed on an area of relevance provides an opportunity to search more broadly than is practical with a bibliographic database, as the number of results is likely to be much smaller. For example, for a series of reviews relating to the transition between inpatient mental health and community and care home settings, we found separate searches with the terms ‘hospital’ or ‘psychiatric’ specific enough to identify a small number of records on some websites, but too generic to use for others.

Schucan-Bird and Tripney [28] describe separate approaches used for searching websites of organisations, subject specialists, research funding bodies and Google for a large systematic literature search. They adopted a general approach for websites of organisations, which involved browsing all items listed under one section where the publications numbered less than 100, and used a search function drawing on a pool of search terms where they found over 100 publications were listed. In contrast, Godin et al. [17] describe a different approach where they searched a website database or used the search function and hand-searched where these functions were not available. Mahood et al. [18] describe an approach where they compiled a pool of search terms, customised the search and used controlled vocabulary wherever possible. In several databases, a simple strategy of two key terms that could be truncated was used in addition to a full or modified longer set of search terms to ensure a comprehensive search. For online repositories, they used their simple strategy in various fields (title, abstract, where available), with result yields varying from two to over 500 references.

Evaluations on utilising specific resources can guide practice. Glanville et al. [25] studied search approaches for two clinical trials registries, ICTRP and Clinicaltrials.gov, and found single-concept searches in the basic interfaces to be the most reliable. Haddaway et al. [24] investigated approaches for searching Google Scholar, which only displays the first 1000 references of a search; they found that title searches enabled discovery of more grey literature (conference proceedings, theses, reports) than full-text searches. They also found that these types of publications occur later in the ranked list of results than academic journal papers.

#### Screening and information management

Once a resource is searched, it must be decided which results should be saved for screening (or sifting) for relevance against the eligibility criteria for a review. This stage also involves recordkeeping to quantify how many literature citations have been processed and the methods used for selecting potentially relevant literature.

There is often no function to export results automatically into citation management tools, so challenges include deciding which literature to keep from each resource, how much screening to carry out within each resource and the level of detail for recordkeeping for preliminary screening of results. Manually transferring all the results is possible, but usually some boundaries need to be set on what is transferred; otherwise, it is an inefficient exercise to collect all references, regardless of their relevance, so they can be screened in a systematic way. Furthermore, transferring all results could promote bias in only undertaking highly focused searches, so that the results manageable, and hinder expansive browsing and iterative searches. We expect that preliminary screening within the website is necessary so that only items that are relevant are saved for further examination. In this way, it can be helpful to consider searching and screening as continuous and iterative. Conceptually searching and screening are on a continuum, as they are both aimed at narrowing a collection of research into those most relevant to answer a review question. However, it is important to consider how much transparency is needed in describing the screening of studies at source.

In our opinion, an efficient way to view screening on websites is to seek out only the items that are of likely relevance to a review and record the number collected for formal screening against eligibility criteria, rather than recording the decision made about the relevance of every citation that is encountered. This approach focuses on describing the literature found. It is possible to have some transparency in assessing the results. For example, indicating whether the literature was assessed for relevance on the basis of the title alone, title and abstract or full text. Where partial lists are browsed, an indication of how that list was organised should be provided (for example, scanning the first 100 items by relevance). Reporting and screening may vary between each resource, as results might be displayed in different ways (for example, a list, a selected quantity displayed by relevance, or chronologically). Where results are scanned by relevance, it depends on user-judgement of how many is appropriate to scan for that particular source.

It is not always possible to know the total number of items scanned in a list (without manually counting). However, in all situations, it is possible to record how many items were retained for further consideration to the literature review. In our opinion, if there is sufficient information on the method used for searching and screening for studies on a website, the precise number of records scanned is of relatively low importance. Table 4 provides an example of documenting this approach. There is space to record whether automated exporting was used, to describe how many results from each search were saved for further screening, how the items were assessed and to record how many items were scanned (if known). However, this approach may not be acceptable to some review teams: Rader et al. [29] observed that in documenting records from non-database sources, some information specialists find it practical only to report those that will be put forward to the review team for screening; others prefer to be precise in reporting every record, even if only a portion of these are included in the final report.

**Table 4:** Example of recordkeeping for initial screening and information management

Name of resource	State Y if automated exporting in RIS/XML	If no automated exporting available:			Additional notes
		No. of promising documents	Number scanned	Approach to screening, e.g. title, then abstract/full text OR first 100 ranked by relevance	
Alzheimer’s Society Research https://www.alzheimers.org.uk/research		0	n/a	Title	
		0	n/a	Title	
		12	70	Title	
		1	69, 23, 43 items from the searches	Title	
Rehabdata		21	37	Title	

Screening at source may be particularly time-consuming depending on the type of literature or if the results contain a lot of relevant records that have already been identified from other searches. Mahood et al. [18] observe that with non-journal literature, it is sometimes difficult to judge relevance based on titles and abstracts due to missing citation information or abstracts. They also observe that duplicate references from Google and Google Scholar can be difficult to recognise due to different citation formats and missing citation information. One approach we have used with Google Scholar is to individually export the results into a citation management tool and duplicate check these against records collected elsewhere, leaving a smaller number of citations to examine. Godin et al. [17] use a bookmarking system within their web browser to avoid identifying the same record twice as the URL of previously bookmarked pages are starred.

Where an internet search engine yielding vast numbers of results in a ranked order, one must decide how many results to screen. This may be informed by the results returned and then screening to saturation (for example, scanning until no more relevant items are identified on a page or on a following page). Based upon a utility analysis of Google Scholar for seven reviews in environmental science, Haddaway et al. [24] recommend looking at the first 300 results in Google Scholar for academic literature, screening well beyond this to find relevant grey literature and they advocate the use of tools to gain a snapshot of the first 1000 results.

### Advances in technology

Advances in technology are likely to increase options for automating website searching, data and document retrieval and recordkeeping in the future. Automated logging tools that store search history, browsing patterns and saving of content have the potential to assist the entire process. For example, the Open Source Internet Research Tool (http://osirtbrowser.com/) provides screen captures, a log of the locations and time a webpage was browsed, fields for the user to record their own notes and also facilitates file management. Other tools may be helpful when searching websites. For example, bookmark management and screen-clipping tools can be used to save and organise information. Web-scraping tools, such as import.io, which extract data from websites, are becoming accessible to users without programming expertise, and may be a useful application for exploring and managing the content examined from some websites.

These tools have the potential appeal for reducing manual recordkeeping. However, the elements logged need to be meaningful, and their use could possibly affect the processes of reflection and iteration encountered in searching. Perhaps these processes illustrate two contrasting approaches: (a) targeted hand-searching such as browsing and manual assessment and retrieval of relevant items and (b) capturing large quantities of studies, including many irrelevant studies, using automated tools and filtering for relevance using text-mining technologies. We expect a hybrid of both approaches would co-exist. Overall, their performance in comparison with manual browsing, searching and document retrieval from individual websites needs to be considered.

### Transparency, accountability and reproducibility

Recordkeeping provides transparency, accountability and reproducibility of the process to varying extents. Transparency is achieved by recording brief information on a resource searched, how and when it was searched, the approach to screening for relevant literature and the number of relevant items saved for further assessment. For many systematic reviews, we have captured the information as an internal record of what was done, though it is potentially available to a wider audience. Publishing fuller details of the search process may be appropriate where there is more reliance on website sources than on traditional bibliographic databases. Accountability is achieved to some extent by having a rationale for the resources searched and having a record of how the search was carried out. This rationale is influenced by several factors, such as the knowledge and skills of the searcher, and time and resource constraints under which a review takes place. A related aspect is that recordkeeping helps searchers consider how they are searching, thereby improving the quality of the search undertaken. These factors are largely hidden from an independent reader of a systematic review, but influence the conduct of searches. Reproducibility of searches is achieved to a limited extent whereby the general approach taken could potentially be replicated. However, clear, systematic and replicable approaches to searching might not lead to replicable results, as observed by Adams et al. [30]. Within each resource, reproducibility is limited as the content of the resources, search functionality and underlying search mechanisms are not static. Furthermore, how they are searched depends on the different perspectives and skills of a user. Limitations for reproducibility do not outweigh the advantage of searching these resources, and we suggest a greater emphasis on transparency and accountability is more appropriate.

## Conclusion

We present a process of systematic website searching in relation to problems encountered in making the process transparent. The methods of website searching may differ between systematic review groups; however, we suggest that it is more important to have a considered rationale for the process, taking into consideration the aims and objective for each review rather than specifying a uniform method. The framework of planning the search, executing the search and screening and information management provides both structure and flexibility to this approach. Recording key elements of the process facilitates reflection and consideration and helps researchers work through some of the challenges of searching websites. New technologies offer potential for automating the browsing, searching and document retrieval processes from websites, which are likely to influence current practice. Despite such advances, the core principles of systematic literature review methods remain that require transparency, accountability and reproducibility. There is a need to raise awareness with website providers and organisations to make their empirical research (and other relevant literature) more accessible to systematic reviewers. We have made some suggestions in Table 5. We hope this discussion will help improve methods in this area.

**Table 5:** Recommendations for website providers

• Consider how users can discover the most relevant content
• Provide instructions or labels for locating research
• Separate empirical research from opinion pieces and guidance tools
• Make all search results available
• Inform users how results are displayed (e.g. relevancy ranked, date published, date added)
• Provide functionality to export citations into citation management tools
